# Large-scale *in vitro* production, refolding and dimerization of PsbS in different microenvironments

**DOI:** 10.1038/s41598-017-15068-3

**Published:** 2017-11-09

**Authors:** Maithili Krishnan, Geri F. Moolenaar, Karthick Babu Sai Sankar Gupta, Nora Goosen, Anjali Pandit

**Affiliations:** Leiden University, Leiden Institute of Chemistry, Gorlaeus Laboratories, Einsteinweg, 55 2333 CC Leiden, The Netherlands

## Abstract

Plants adapt to fluctuating light conditions by a process called non-photochemical quenching (NPQ), where membrane protein PsbS plays a crucial role and transforms a change in the pH-gradient across the thylakoid membrane under excess light conditions into a photoprotective state, leading to de-excitation of antenna chlorophylls. The PsbS activation mechanism is elusive and has been proposed to involve a monomerization step and protonation of specific residues. To elucidate its function, it is essential to produce PsbS in large quantities, stabilize PsbS in a membrane-mimicking environment and analyze its pH-dependent conformational structure. We present an approach for large-scale *in-vitro* production and spectroscopic characterization of PsbS under controlled, non-crystalline conditions. We produced PsbS of the moss *Physcomitrella patens* in milligram quantities in *E*. *coli*, refolded PsbS in several detergent types and analyzed its conformation at neutral and low pH by Dynamic Light Scattering and NMR spectroscopy. Our results reveal that at both pH conditions, PsbS exist as dimers or in apparent monomer-dimer equilibria. Lowering of the pH induces conformational changes, destabilizes the dimer state and shifts the equilibria towards the monomeric form. *In vivo*, a similar response upon thylakoid lumen acidification may tune PsbS activity in a gradual manner.

## Introduction

Photosynthesis is the process of converting light energy to chemical energy carried out by all plants and photosynthetic algae. One of the major challenges faced is the excess of light during peak time of the day^1^. Light changes rapidly due to variations in weather conditions, geographic locations and seasonal fluctuations. High light intensity causes over excitation of the photosynthetic antenna and overload of the electron-transport chain producing damaging reactive oxygen species (ROS). Plants have evolved several photo protection mechanisms to survive these ROS. One such mechanism allows plants to adapt to surplus light by dissipating the excess light energy as heat^2^. This process is termed as non-photochemical quenching (NPQ) which takes place primarily in the antenna associated with photosystem II (PSII) complex located in the thylakoid membrane of chloroplasts^3^. The most important aspect for NPQ is the existence of a proton gradient across the thylakoid membrane, which senses the photosynthetic state during varying light conditions. During low light conditions, the proton gradient is low and the photosynthetic antenna is in a light harvesting state. However, during high light, the proton gradient increases until acidification of the internal thylakoid compartments reaches a threshold value triggering NPQ^4^.

The discovery of the membrane protein Photosystem II subunit S (PsbS) made it clear that this protein has a central role in sensing the thylakoid luminal pH and activating a series of complex structural rearrangements that lead to chlorophyll (Chl) de-excitation in the antenna, which is the basis of NPQ^5,6^. The photosynthetic subunit PsbS belongs to the light harvesting complex (LHC) protein superfamily^7^. It has a molecular weight of 22 kDa and has four transmembrane helixes in its structure, unlike all other members of the LHC family. Studies proved that PsbS is a prime requirement for excited-state quenching (qE) upon proton accumulation *in vivo* ^8,9^. PsbS doesn’t bind specific Chl pigments and acts as a pH sensor to trigger structural changes in Photosystem II- Light Harvesting Complex II (PSII-LHCII) super-complexes leading to qE and NPQ^10,11^. Recent studies focusing on PsbS interaction partners showed that *Physcomitrella patens* PsbS associates with one of the LHCII trimer (Lhcb1) proteins^12^ for PsbS of higher plants, enhanced interaction with Lhcb1 antenna complexes was observed under high-light conditions^13^, and increased binding of PsbS to minor antenna complexes was observed with the combination low pH and zeaxanthin^14^. Overexpression of PsbS in Tobacco plants, together with xanthophyll-cycle enzymes, led to increase in qE along with accelerating NPQ relaxation, which significantly increased the quantum yield of CO_2_ assimilation^15^.

Mutational studies on glutamate residues in the lumen region of PsbS, E122Q and E226Q, have shown to severely influence qE, indicating that they are critical for the function of PsbS^16^. Protonation of the two glutamates were proposed to induce a dimer to monomer transition, which is suggested to be the first step occurring in PsbS activation^17^. The recent crystallography structure however shows that PsbS forms a stable dimer under low-pH conditions^18^. *In vivo* and *in vitro* studies have been carried out on PsbS to understand its underlying mechanism in the regulation of NPQ and interaction with antenna proteins^10,14,16,19^. It is however challenging to determine the molecular function of PsbS, due to its lack of specific-bound pigments, its low production in plants and difficulty of protein purification. Few studies performed *in vitro* synthesis of PsbS^10,11,17,19^, producing detergent-refolded PsbS in moderate quantities, and PsbS has been refolded in liposomes together with LHCII to study its quenching mechanism *in vitro* ^19,20^.

In this work, we established a protocol to produce and refold recombinant *Physcomitrella patens* PsbS in milligram quantities that can be used for thorough structural characterization by NMR spectroscopy or X-ray diffraction, which techniques are highly demanding in terms of required protein quantities. Since PsbS in isolated form lacks a functional assay, we carefully analyzed its refolding in different types of detergent that mimic the membrane micro-environmental conditions, and demonstrate successful refolding using various detergent types. To the best of our knowledge, we are the first to report the association of recombinant PsbS into native-like dimers, and analyzed the oligomeric states and conformational structure of PsbS by a combination of SDS-gel analysis, dynamic light scattering (DLS), size exclusion chromatography (SEC), diffusion-ordered spectroscopy (DOSY) and hetero nuclear single quantum coherence (HSQC) NMR spectroscopy. Experiments were carried out at neutral and low pH, mimicking the inactive and active state of PsbS, to understand how PsbS folding and assembly is controlled by the pH environment.

## Results and Discussion

### Optimization of PsbS overproduction and refolding

**Figure 1:**
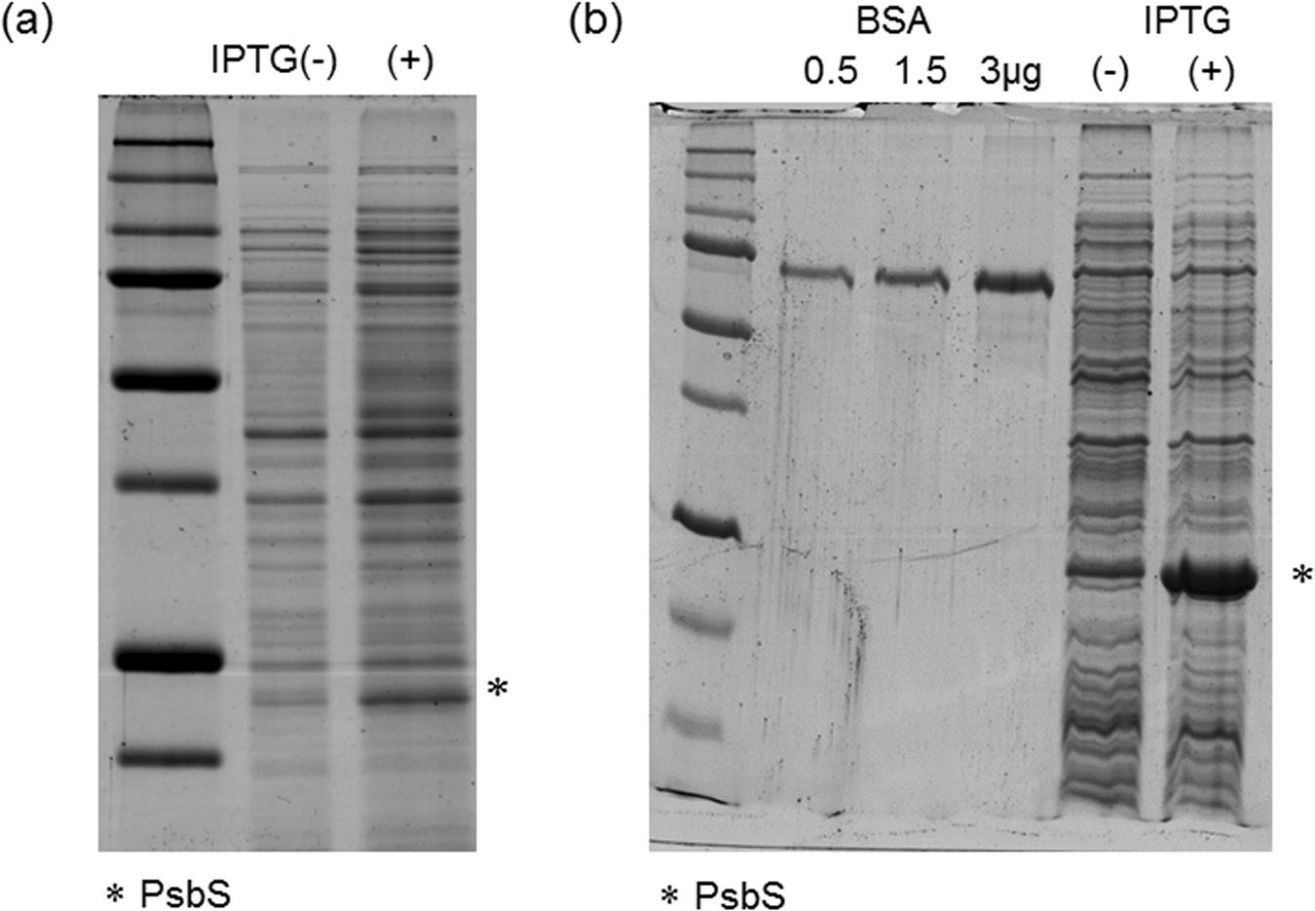
Overproduction of PsbS. (**a**) PsbS overexpression using the pETite vector (yield: ~9 mg from 200 mL cells); (**b**) PsbS overexpression using the pExp5 vector (yield: ~25 mg from 200 mL cells).

The first goal of our study was to optimize production of PsbS in *E*. coli cells, to have a protocol by which PsbS could be produced in milligram quantities. We improved the overproduction of PsbS by optimizing the choice of expression vector and by modification of the purification protocol. In Figure 1a, IPTG (+) shows the overexpression of PsbS using the vector pETite. About 9 mg of PsbS was expressed from 200 mL of culture solution (i.e. ~45 mg PsbS/L). With the new vector system pExp5, PsbS overexpression was increased considerably (Fig. 1b, IPTG+). We estimated the PsbS expression yield by comparing the intensities of the PsbS bands to the intensity of a known concentration of BSA protein. We obtained about 25 mg PsbS from 200 mL of cell culture (i.e. ~125 mg PsbS/L), giving almost a three-fold increase in production compared to expression using the pETite vector.

**Figure 2:**
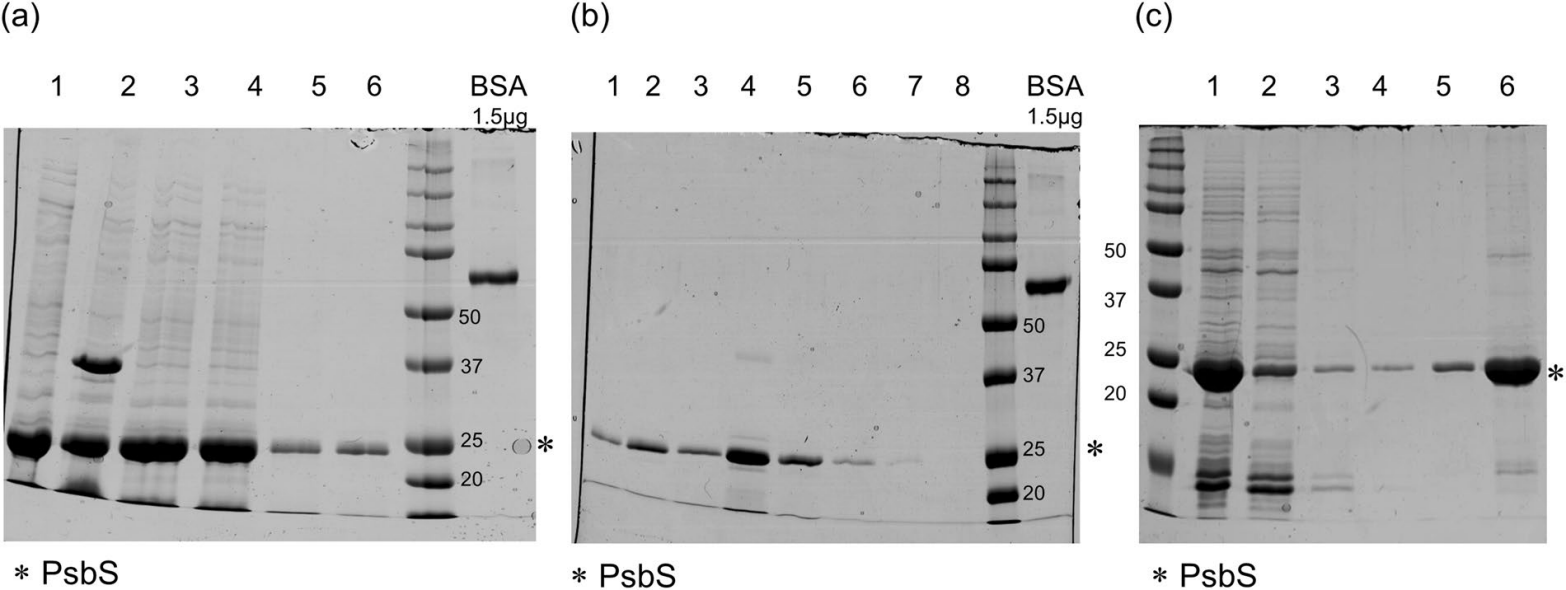
Purification of PsbS from inclusion bodies using nickel-affinity column or urea-wash protocol. (**a**) Inclusion bodies pellet P4 (lane 1) containing 19 mg of PsbS was solubilized in urea buffer containing 0.5% LDS. Pellet (lane 2) and supernatant (lane 3) were obtained after centrifuging at 10000xg for 10 minutes. Supernatant was loaded on the nickel affinity column. The loss fractions of PsbS are shown as the flow through (lane 4) and wash (lane 5,6), and together give a loss of ~14 mg PsbS; (**b**) Nickel-affinity column purified elution frations of PsbS (1 µl loaded on gel). Elution fraction 3 (0.7 µg/µl), fraction 4 (1 µg/µl) and fraction 5 (0.7 µg/µl) were further used to refold PsbS (lane 3,4,5). In total, the collected fractions contained ~3.6 mg of PsbS out of 19 mg starting material. (**c**) UP-wash protocol; Lane 1: About 3.2 mg of PsbS from the inclusion bodies pellet was dissolved in buffer containing 8 M urea. Several washes of urea buffer were carried out (Lane 2, 3, 4). Lane 5 is washing step of pellet with 8 M urea buffer with 0.05% LDS. The last wash step was carried out using urea buffer with 0.5% of LDS to dissolve all the PsbS from inclusion bodies (Lane 6). The concentration of PsbS in the 0.5% LDS buffer was ~2 µg/µL (2.5 µL sample loaded on gel). In total ~2 mg of PsbS was present after urea-wash purification from 3.2 mg starting material.

To further improve the production of PsbS, we wanted to reduce the loss of protein during its purification steps. The basic purification of inclusion bodies containing PsbS from the cell pellets is shown in Supplementary Fig. S1, using detergent buffer and Triton buffer wash. The final inclusion bodies pellet (P4) contained about 19 mg of PsbS, which was used as a starting material for PsbS purification. Figure 2a and b demonstrates that with standard nickel-affinity column purification using the N-terminal His-tag, significant loss of protein occurs. In 2a, the inclusion bodies pellet was first dissolved in 8 M urea in phosphate buffer pH 8.0 (UP-buffer) containing 0.5% Lithium dodecyl sulfate (LDS) and centrifuged to give pellet and supernatant. Supernatant was loaded on the nickel-affinity column. During the column purification process ~14 mg of PsbS was discarded in flow through from the 19 mg starting material. The overall efficiency of the column method was estimated to be ~19%, with about 80% of the protein lost in the flow through or other eluted fractions. To reduce loss of protein during the purification steps, we implemented a new purification protocol that circumvented the use of nickel-affinity column purification. Our new method to purify PsbS was based on the poor solubilty of PsbS in UP-buffer either without LDS or in the presence of 0.05% LDS. After two times washing the pellet with UP-buffer without LDS, the washing was continued several times with UP-buffer containing 0.05% LDS to remove contaminated proteins. Finally, the purified PsbS pellet was dissolved in UP-buffer containing 0.5% LDS (results shown in Fig. 2c). The starting material for purification contained about 3.2 mg of protein (lane 1). Several washes with UP buffer showed removal of background proteins (lane 2, 3, 4). The final amount of purified PsbS was about 2 mg, by which the efficiency of this method was 60%, compared to 19% using the nickel-affinity column.

Summarizing, the combination of using pExp5 expression vectors and the UP-buffer wash method for purification gave almost three-fold increase in overproduction and another three-fold increase of purification yields, by which our modifications resulted in about 9-fold increase of PsbS production. A possible reason for loss of PsbS during nickel-affinity column purification could be that proteins are unfolded in the presence of urea leading to poor binding to the column. Moreover, the presence of 2% LDS in the nickel column can interfere with the affinity for PsbS, making the nickel-column protocol quite tedious for large-scale purification. The UP-wash method on the other hand can be easily applied for obtaining large quantities of PsbS, circumvents the use of column purification and does not require engineering of a His-tag. To test the applicability of our purification method for other LHC proteins, we applied our UP-wash protocol to purify the Lhcb1 protein of *Arabidopsis thaliana*, a protein homologue to PsbS and one of the polypeptides forming trimeric Light-Harvesting Complex II (LHCII) (Supplementary Fig. S2). The UP-wash method applied to Lhcb1 gave yields that were comparable to PsbS for purification of the protein from inclusion bodies, confirming that the method is extendable to other proteins of the LHC family.

**Figure 3:**
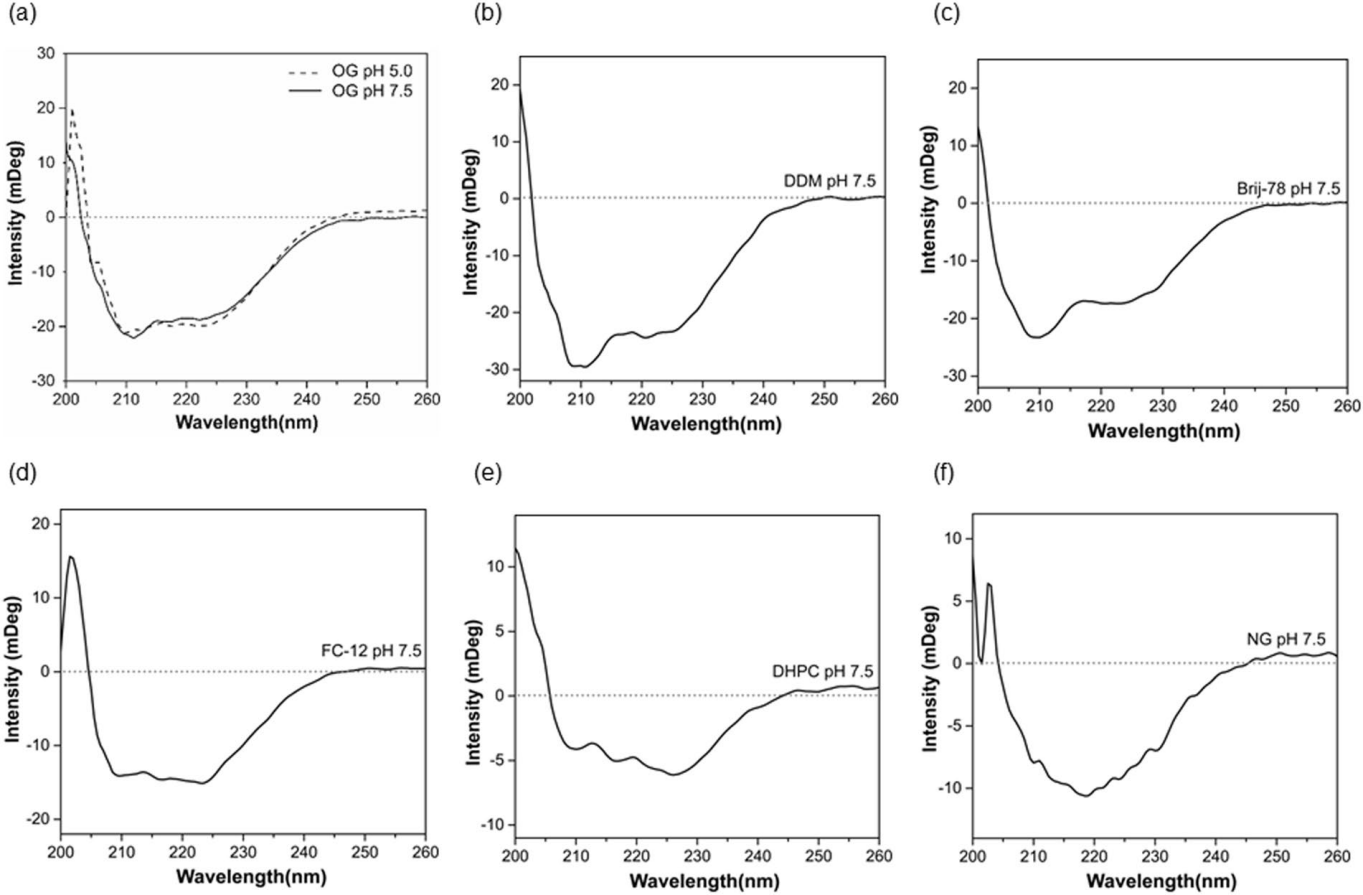
Circular dichroism spectra of PsbS refolded in various detergent buffers. CD spectra of PsbS refolded in buffer containing (**a**) OG at pH 7.5 (solid line) and at pH 5.0 (dashed line) (**b**) DDM (**c**) Brij-78 (**d**) FC-12 (**e**) DHPC and (**f**) NG.

The second aim of our study was to choose an optimal detergent for refolding of PsbS. Refolding of recombinant plant PsbS in detergent micelles has been shown successful using *n*-Octyl-*β*-D-Glucopyranoside (OG) or *n*-Dodecyl-*β*-D-Maltoside (DDM)^10,19^, and the crystal structure of spinach PsbS was obtained from PsbS purified in *n*-Nonyl-*β*-D-Glucopyranoside (NG) buffer^18^. In addition to these glucoside-based detergents, we tested refolding in the phosphocholine detergents *n*-Dodecyl phosphocholine (FC-12) and 1,2-diheptanoyl-*sn*-glycero-3-phosphocholine (DHPC), which have been used successfully as membrane mimics for solution NMR^21^, owing to their short chains and small micelle sizes. Further, we tested refolding in Brij-78, which is an oxyethylene-polymer based detergent that has suitable properties for *in vitro* expression and refolding using cell-free expression systems^22^. Figure 3 shows circular dichroism (CD) spectra of refolded PsbS using various detergents. PsbS was successfully refolded in OG, DDM, Brij-78 and FC-12, as seen from the CD spectral shapes that are representative of helical structures in Fig. 3a–d. However, refolding in DHPC (e) or NG (f) would significantly reduce the amount of helicity. Table 1 presents a CD spectral component analysis giving an estimate percentage of the helix, antiparallel, parallel or turn structures present in the refolded PsbS. Using the homologous crystal structure of PsbS from *Spinacia oleracea* ^18^, for the *Physcomitrella patens* PsbS amino-acid sequence 47% α-helical structure is predicted^18^ closely matching with the CD analyses except for NG and DHPC. For NG, this is remarkable since this detergent was successfully used for crystallization of native PsbS. It is interesting to notice that for PsbS refolded in OG, DDM, Brij-78 and FC-12, which all gave samples with similar percentage of helicity, the percentage of sheet structure differ for the various detergent types. Supplementary Fig. S3 shows a homology structure of *Physcomitrella patens* PsbS. The structure contains two small anti-parallel sheet stretches at the stromal site. In addition, the stromal site contains a large loop segment that is not resolved in the crystal structure, and contains the N- and C-terminal stretches that are only partly resolved. At the luminal site, the trans-membrane protein helices are connected via short connected loops that contain the proposed Glu active residues and a short helix segment. We suspect that the non-helical protein sites can adapt different folds depending on the detergent micelles in which the protein is solubilized. The CD spectrum of our recombinant PsbS in DDM looks very similar to the published CD spectrum of native plant PsbS in DDM^23^, with characteristic peaks at 208 nm and 222 nm, of which the 208-nm peak is very pronounced. The 208-nm peak is less pronounced in the CD spectrum of PsbS refolded in OG, and this spectrum of PsbS in OG buffer looks very similar to the CD spectrum reported by Wilk *et al*.^19^, who refolded plant PsbS in OG micelles. The CD spectral differences for PsbS solubilized in various detergents likely reflect structural differences that are induced by the detergent micellar shapes. DDM micelles form oblate structures, whereas OG and FC-12 micelles form prolate structures^24^ and Brij-78 is a polymeric surfactant that forms random-coil arrangements. Only small differences were observed comparing the CD spectra at neutral and low pH. Figure 3a contains the CD spectra of refolded PsbS in OG, in pH 5.0 and 7.5 conditions. In both pH conditions, PsbS adopts a ~50% α-helical structure according to the CD component analysis in Table 1. There are minor changes in the non-helical spectral contributions that are however small compared to the structural variation this is observed for different detergent types. In conclusion, several detergents were found to be suitable for refolding of PsbS. We selected OG and FC-12 for further characterization, since both detergents have small micellar sizes, which is favorable for size-determination analyses.

**Table 1:** CD Secondary-structure analysis of PsbS in various detergent buffers.

Secondary structures (%)	OG pH 7.5	OG pH 5.0	DDM pH 7.5	Brij-78 pH 7.5	FC-12 pH 7.5	DHPC pH 7.5	NG pH 7.5
Helix	51.8	50.8	51.4	42.3	49.6	17.2	25.2
Antiparallel	11.5	0	30.5	17.5	1.1	10.1	10.6
Parallel	0	9.8	0	0	0	0	9.8
Turn	8.6	6	2.3	8.4	8.1	21	14.5
Others	28.1	33.8	15.8	31.8	41.2	51.6	39.8

### The oligomeric state of detergent-refolded PsbS at low and neutral pH

Native PsbS exists as dimers at neutral pH and as monomers at low pH according to^13,17^ or may cluster into larger aggregates under NPQ conditions^14^. Fan *et al*. however determined the structure of a dimeric PsbS at low pH, and PsbS dimer states at neutral and low-pH conditions were observed by SDS-page analysis^18^. Although SDS-page analysis can provide good estimates of molecular weights, protein oligomeric states may alter after sample preparation in SDS buffer and subsequent heating. Using chemical crosslinking, protein oligomeric states are stabilized, however cross-linking efficiencies itself can be pH-dependent. We used three additional methods to determine the sizes of refolded PsbS directly in detergent solutions. Dynamic Light Scattering (DLS) was applied as a rapid method to determine hydrodynamic size distributions of PsbS in detergent solution. Size exclusion chromatography (SEC) was carried out to determine the oligomeric states of PsbS. Diffusion ordered spectroscopy NMR (DOSY NMR) was applied as alternative to DLS to determine protein translational diffusion constants, from which the protein-micelle hydrodynamic sizes were estimated. DOSY NMR has the advantage over DLS that the proteins are probed directly via the amide protons, whereas DLS detects both empty and protein-containing micelles.

**Figure 4:**
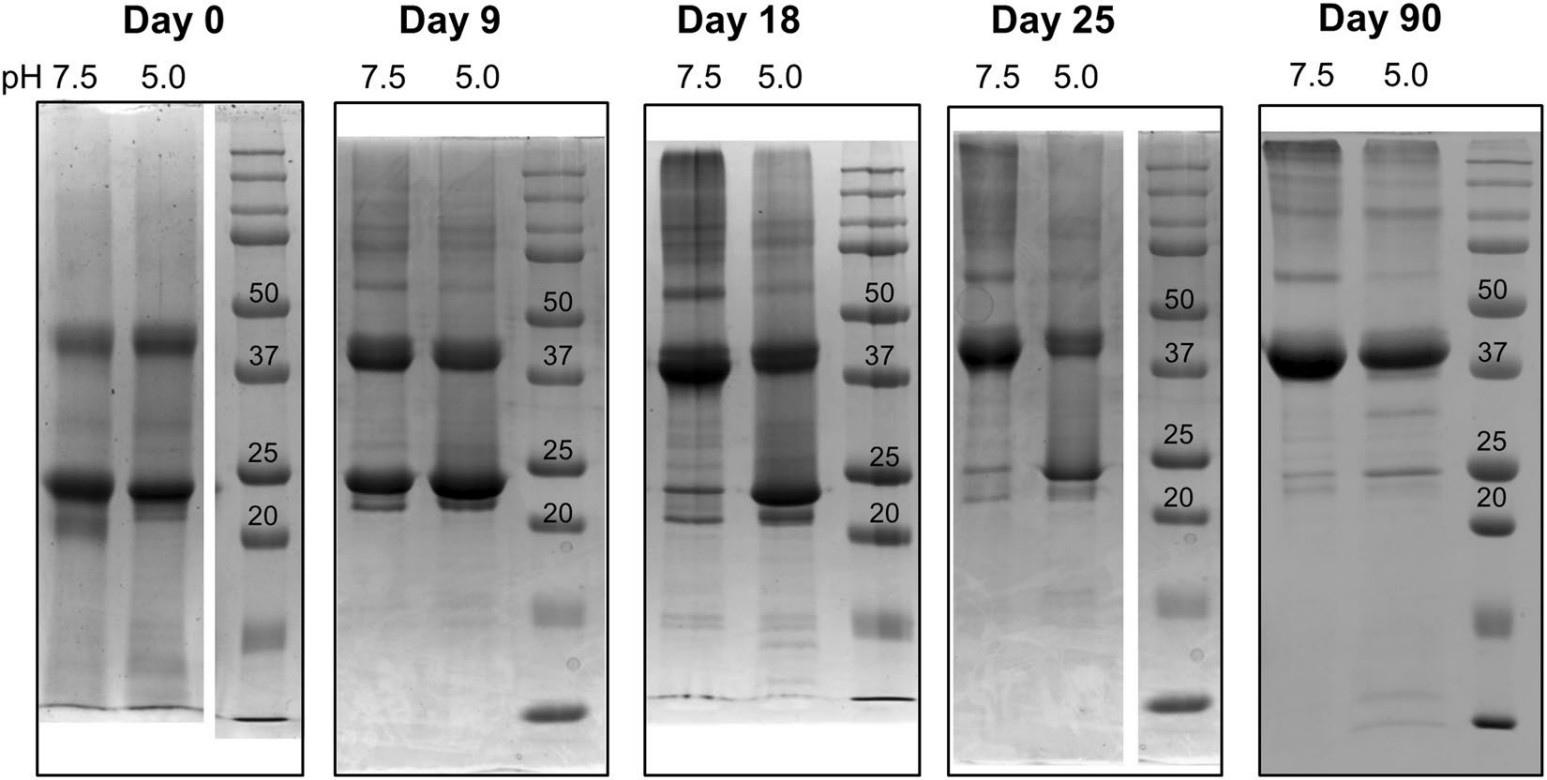
SDS-page gel analysis of samples collected at different days after PsbS refolding. SDS-page gel graphs of PsbS collected at various days after its refolding in FC-12 detergent. Samples were not boiled before loading on gel.

To investigate the oligomeric states and stability of PsbS, we carried out SDS-page gel analysis experiments, where PsbS samples were analyzed between zero and 90 days after PsbS refolding in FC-12 buffer. We found that the dimer states were resistant to SDS solubilization when the subsequent boiling step was omitted (Supplementary Fig. S4). Therefore, we omitted the standard boiling step for the SDS-page analysis. In Figure 4, SDS-page gels for PsbS in FC-12 at pH 7.5 and at pH 5.0 are shown respectively, collected at different days after detergent refolding (defined as Day 0). At day 0, PsbS forms mixtures of monomers and dimers both at neutral and low pH. On a time scale of weeks, the dimer content of the samples slowly increased (Day 9 and 18). Interestingly, this process appeared to be retarded at low pH. At Day 25, the pH 7.5 sample predominantly shows as a dimer band, whereas the pH 5.0 sample contains monomer and dimer bands of similar intensities. After three months (Day 90), PsbS dimers were the most dominant observed form under both pH conditions. In addition to monomer and dimer forms, the gel graphs show that PsbS samples contained fractions of larger oligomers. From the time-dependent analysis, we can deduct that dimeric PsbS is the most stable state at both pH conditions. It is however surprising that it takes several weeks to reach this state. It is possible that PsbS dimers slowly associated into larger aggregates that were not in equilibrium anymore with the monomers. If the aggregates were not resistant to SDS solubilization, the incorporated proteins would appear as dimers on SDS-page gels. It is also possible that FC-12, which is a zwitterionic detergent that is known to prevent protein-protein interactions, retards the association of detergent-solubilized monomers into dimers. The slow observed process of dimerization is informative as it differentiates between neutral and low-pH conditions. The retarded dimerization process at low pH suggests that the PsbS dimers are destabilized, or that there is a larger energetic barrier for their association. In time, solutions also started to form visible sediments and accumulate soluble aggregates. Therefore, the following experiments were performed within a week after PsbS detergent refolding.

**Figure 5:**
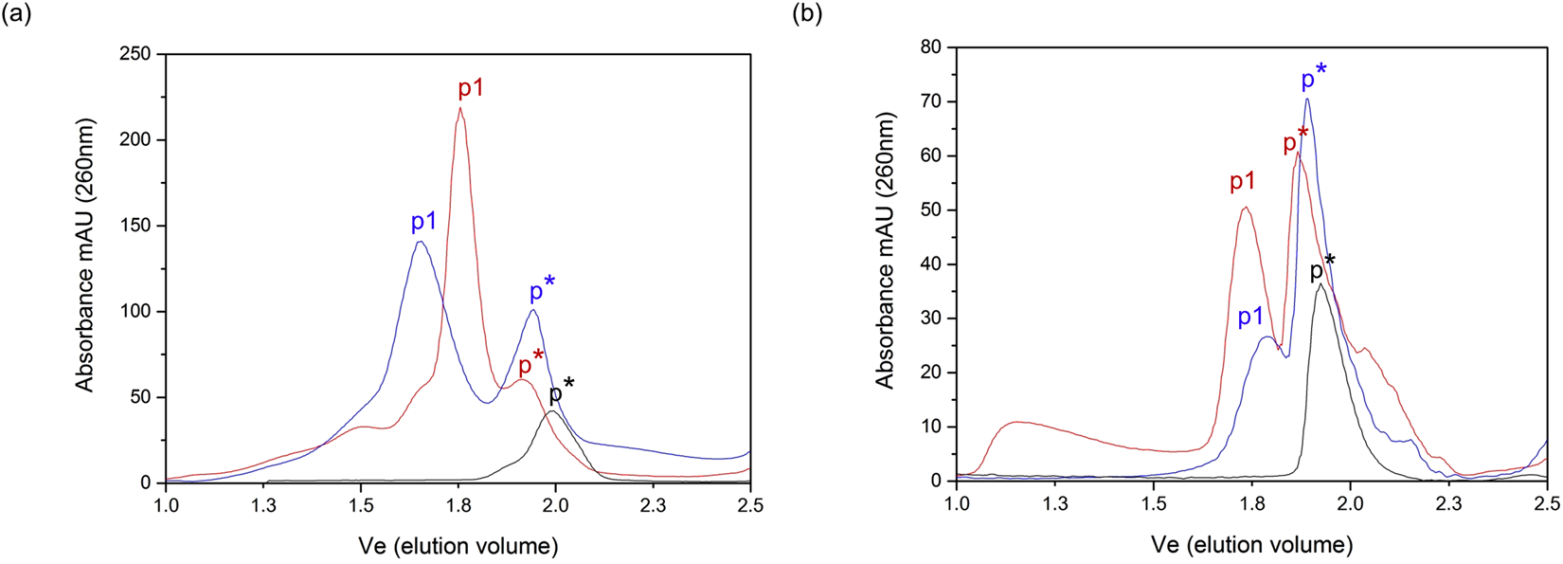
Size exclusion chromatography of PsbS in FC-12 and in OG. SEC chromatograms of (**a**) PsbS in FC-12 at pH 7.5 (red) and at pH 5.0 (blue); (**b**) PsbS in OG at pH 7.5 (red) and at pH 5.0 (blue). Black lines correspond to control experiments with detergent only, FC-12 (**a**) or OG (**b**). The p* peaks appear at similar elution times as the detergent micelles and are considered to be empty detergent micelles, while the p1 peaks are the PsbS fractions.

To analyze the oligomeric states of refolded PsbS in detergent buffer conditions without SDS, PsbS samples were analyzed by SEC. Figure 5 shows the SEC graphs for PsbS in FC-12 (5a) and OG (5b) at pH 7.5 (red) and pH 5.0 (blue). In addition, control experiments were performed injecting 10% of FC-12 or 10% of OG detergent buffered at pH 7.5 (black lines). The detection wavelength was set at 260 nm to be at the maximum absorption of phenylalanine (Phe) since *Physcomitrella patens* PsbS has 21 Phe residues but contains no tyrosine or tryptophan. Under all conditions, two prominent peaks assigned p* and p1 were observed, together with weak peaks representative of larger aggregates. In the control experiments, OG and FC-12 eluted from the column at similar times as the p* peak of PsbS. We assume that the p* peak contains empty detergent micelles. In agreement, a SEC experiment of PsbS in OG (pH 7.5) with the detection set at 214 nm does not show the p* peak (Supplementary Fig. S5). FC-12 and sodium-acetate buffer solutions had significant intrinsic absorption at 214 nm and therefore SEC with 214 nm detection was not applicable. The SEC estimated detergent micelle sizes were ~40 kDa for FC-12 and ~50 kDa for OG (Supplementary Tables S1 and S2). Considering a micelle size of ~40 kDa for FC-12, and p1 corresponding to ~90 kDa at pH 7.5, the p1 peak in Figure 5a would match with the size of PsbS dimers (protein size ~44 kDa). For PsbS in OG, p1 (~100 kDa) also would match with the size of PsbS dimers considering OG micelle sizes of ~50 kDa. At low pH, solutions of PsbS in OG contained visible sediments. In the SEC run of PsbS in OG, the shoulder of soluble aggregates at the void volume between 1-1.3 Ve (Fig. 5b, red) disappears at low pH (5b, blue), which is a strong indication that soluble aggregates at pH 7.5 formed sediments at pH 5.0. Because of the low intrinsic (Phe) absorption of *patens* PsbS, and incompatibility with 214 nm detection for PsbS in FC-12 and for PsbS in OG at low pH, we cannot exclude that in the latter solutions PsbS monomers were present that were obscured by the p* detergent peaks. Our finding that PsbS in FC-12 migrates faster from the column at pH 5.0 than at pH 7.5, is opposite to the findings of Fan *et al*., who found that spinach PsbS purified in NG would migrate slower at low pH^18^. We suspect that low-pH solutions of FC-12 contained mixtures of dimers and larger oligomers, or that PsbS protonation changes had a different effect on the elution profile due to the zwitterionic character of FC-12.

**Figure 6:**
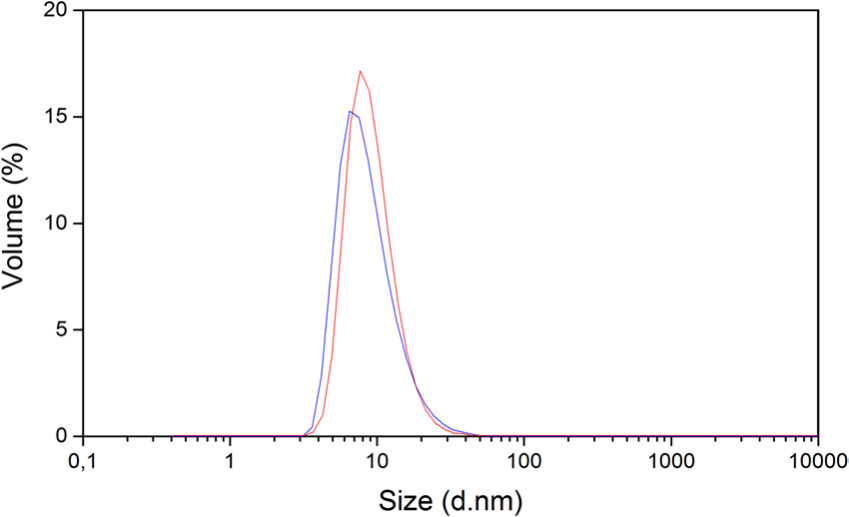
Dynamic light scattering of PsbS in FC-12. PsbS particle size determined by DLS on PsbS refolded in FC-12 at pH 7.5 (red) and at pH 5.0 (blue).

To further analyze the PsbS particle sizes, a DLS size analysis was performed. Figure 6 shows the DLS size distributions for PsbS in FC-12 at neutral and acidic pH. At low pH (blue curve), a small shift of the distribution towards smaller diameter sizes is observed. Since the DLS Volume% intensities increase with the third power of the diameter sizes, a DLS size distribution of a mixture of monomers and dimers will be biased towards the dimer form. We assume that the average DLS diameter sizes reflect the size of the dimers and that the shift at low pH indicates increase of the monomer population, in agreement with the SDS-page analysis. The DLS distributions are tailed on the right side, indicating presence of larger aggregates. For PsbS in OG, the DLS experiments did not produce reliable results due to high polydispersity of the samples (data not shown), indicating that the solutions contained mixtures of monomers or dimers and a significant population of higher aggregates. The DLS results of PsbS in FC-12 do not show a shift into larger oligomers at low pH, indicating that the SEC peak shift at low pH is due to changes in protein conformation or protonation states.

**Figure 7:**
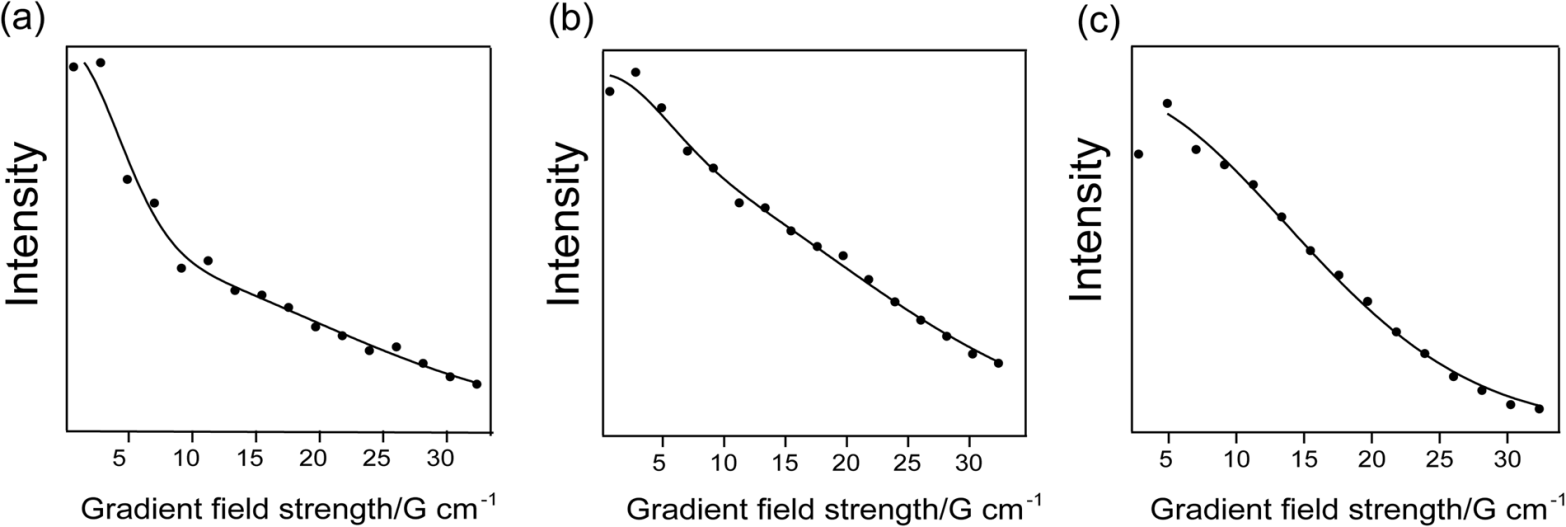
DOSY NMR of PsbS in FC-12. Integrated ^1^H *H*_*N*_ intensities (black circles) versus the gradient field strength of PsbS in FC-12 at (**a**) pH 7.5 and (**b**,**c**), at pH 5.0. The sample in **(c**) is referred to as the monomer sample. Data was fit with a double-exponential fit (black lines) according to Eq. 1 to estimate the diffusion coefficients. Fitted translational diffusion constants were (**a**) *D*_*t*_ = 4.0 • 10^−11^ m^2^ s^−1^; (**b**) *D*_*t*_ = 3.9 • 10^−11^m^2^ s^−1^ and (**c**) *D*_*t*_ = 7.9 • 10^−11^m^2^ s^−1^.

Solutions of PsbS in FC12 and OG buffer were further analyzed by DOSY NMR spectroscopy. In DOSY, NMR signals of different species are separated according to their diffusion coefficient. A series of spin echo spectra is measured with different pulsed field gradient strengths, and the signal decays are analyzed to extract the translational diffusion coefficients for each species. The measured intensity curves against the gradient strength were fit with a double-exponential function (see Eq. 1 Materials & Methods) to determine the translational diffusion coefficients *D*_*t*_. Figure 7a and b show the *H*_*N*_ DOSY curves for PsbS in FC-12, at pH 7.5 (a) and at pH 5.0 (b) together with the double-exponential fits. The slow exponential component contains the actual diffusion coefficient, while the fast decay component arrives from proton exchange at water-exposed *H*_*N*_ sites. Remarkably, the relative amplitude of the fast decay component was larger at pH 7.5 than at pH 5.0. Adding an additional fit component, considering a mixture of PsbS monomers and dimers with different diffusion properties, did not give an additional diffusion coefficient, neither did it improve the fit. In one sample of PsbS in FC-12 at pH 5.0, a much faster intensity decay was observed (Fig. 7c), corresponding to a much larger diffusion coefficient. We tentatively propose that in this particular sample, PsbS monomers were dominant. This sample was freshly prepared and may have contained larger amounts of detergent than the other samples. The protein-detergent solutions had to be concentrated for the NMR experiments, by which the final amounts of detergent present in the samples were difficult to control. The estimated values for *D*_*t*_ are summarized in Table 2, where the sample in 7c is indicated as ‘monomer’. Data of PsbS in OG at pH 5.0 was omitted because the sample sedimented during the DOSY experiment. The diffusion coefficients estimated from the DOSY curves in Figure 7a and b correspond with hydrodynamic diameter sizes of 12.1 (±1.2) nm at pH 7.5 and 12.4 (±1.3) nm at pH 5.0, and for PsbS in OG (pH 7.5) the diffusion coefficient corresponds with 15.2 (±1.5) nm. For the supposed monomeric sample in Figure 7c, the diffusion coefficient corresponds with 6.2 (±1.0) nm. In addition to the *H*_*N*_ intensities, the trimethyl-amino peak of FC-12 and the (4-7) *CH*_2_ peak of OG were used to estimate the translational diffusion coefficients. The diffusion coefficients measured via the detergent peaks correspond with smaller hydrodynamic sizes, because the detergent signals are the average of free detergent, empty detergent micelles and protein-detergent micelles. The diameter sizes determined from the FC-12 trimethyl-amino peak intensities are 9.4 (±1.3) nm (pH 7.5) and 8.7 (±1.2) nm (pH 5.0) respectively, which are very close to the DLS determined average sizes. Indeed, the DLS determined sizes are also the average of protein- and empty detergent micelles that are not resolved as separate peaks in the histograms. The DOSY results do not show significant changes at low pH, except for the one particular sample. This suggests that under both conditions, PsbS predominantly existed as dimers. Decreased stability of the dimers at low pH could make them more sensitive to fluctuations in detergent concentrations.

**Table 2:** Translational diffusion coefficients of PsbS-detergent micelles.

Sample	D_t_ H_N_ (m^2^s^−1^)	D_t_ FC-12 (m^2^s^−1^)	D_t_ OG (m^2^s^−1^)
PsbS in FC-12, pH 7.5	4.0 · 10^−11^ (3.7 · 10^−12^)	5.0 · 10^−11^ (5.2 · 10^−12^)	—
PsbS in FC-12, pH 5.0	3.9 · 10^−11^ (3.8 · 10^−12^)	5.7 · 10^−11^ (5.6 · 10^−12^)	—
PsbS in FC-12, pH 5.0 (monomer)	7.8 · 10^−11^ (6.6 · 10^−12^)	9.8 · 10^−11^ (1.0 · 10^−11^)	—
PsbS in OG, pH 7.5	3.2 · 10^−11^ (1.1 · 10^−12^)	—	3.9 · 10^−11^ (4.2 · 10^−12^)

Estimated *D*_*t*_ values (standard deviation between brackets) based on the DOSY ^1^H NMR *H*_*N*_ intensities (H_N_), on the FC-12 trimethyl-amino peak (FC-12) or on the OG *(4-7) CH*_2_ band (OG).

### PsbS conformational structures at low and neutral pH

**Figure 8:**
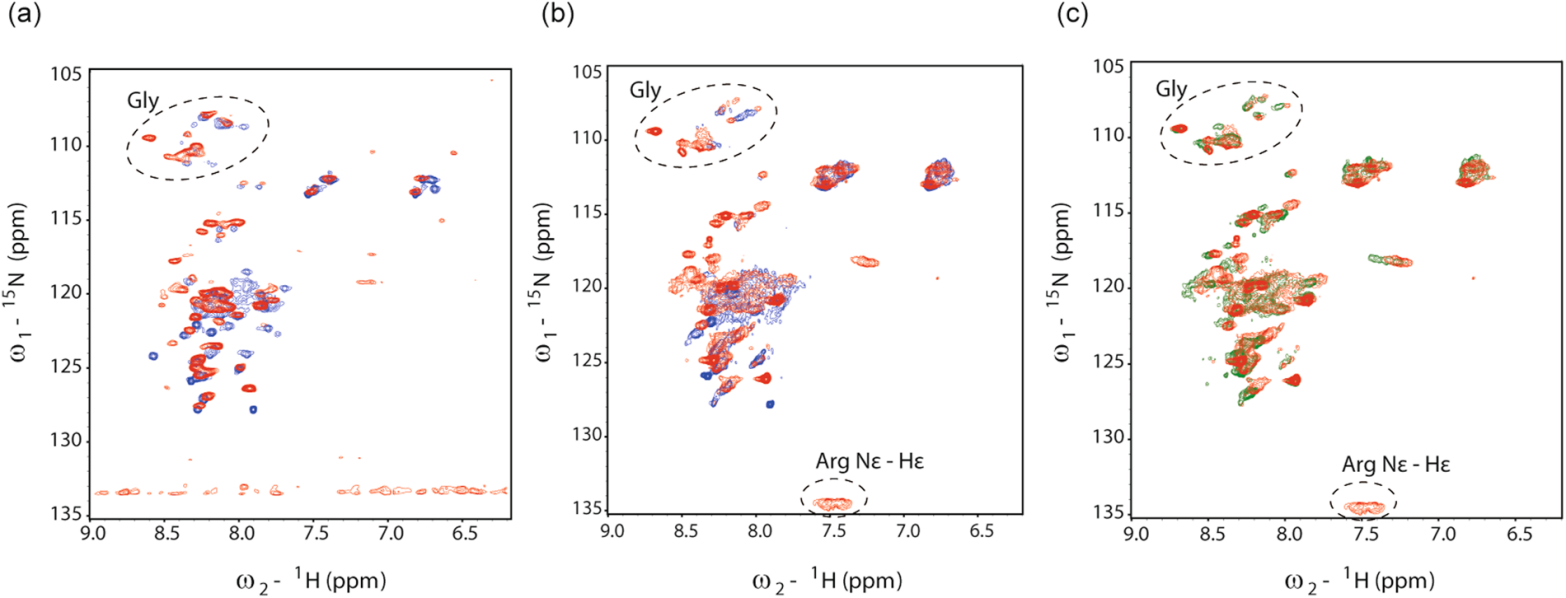
HSQC NMR spectra of PsbS in FC-12 and in OG. (**a**) PsbS in OG at pH 7.5 (blue) and at pH 5.0 (red); (**b**) PsbS in FC-12 at pH 7.5 (blue) and at pH 5.0 (red); (**c**) PsbS in FC-12 at pH 5.0 (red) and the supposed monomerized sample of PsbS in FC-12 at pH 5.0 (green).

To gain further insight in the structure and dynamics of PsbS, ^1^H-^15^N HSQC NMR spectra were recorded of the DOSY samples, that are presented in Figure 8. The Figure 8a presents HSQC spectra of PsbS in OG at low (red) and neutral (blue) pH, and in 8b spectra of PsbS in FC-12 at low (red) and neutral (blue) pH are presented. In Figure 8c, the DOSY samples that were analyzed in Fig. 7b (red) and 7c (green) are compared. The spectra show only a moderate set of resonances with low dispersion, reminiscent of a random-coil structure, which contrasts with the CD spectral analyses that predict ~50% helicity. The contrast is explained if we consider that due to the large protein-micelle sizes, only the mobile protein regions might be resolved in solution NMR, which are the flexible loops, turn and terminal regions that reside in the aqueous phase. Two experimental observations agreed with this notion. An H-D exchange experiment reversibly deleted most of the resonance peaks (data not shown), indicating that these are from water-accessible sites. Second, the DOSY curves contain a fast decay component with considerable amplitude that can be interpreted by fast amide proton exchange with the surrounding bulk water, which does not occur for amides involved in α-helical H-bonds. The HSQC resonance peaks are better resolved for PsbS in OG than for PsbS in FC-12, suggesting that PsbS adopts a more dynamical structure in OG micelles than in FC-12. A comparison of spectra at pH 7.5 and pH 5.0 (Figure 8a and b) show several changes in the peak patterns and more resolved peaks at pH 5.0 (red spectra), including several glycine (Gly) peaks and a folded peak of the arginine (Arg) guanidinium side-chain nitrogens for PsbS in FC-12. The improved resolution at pH 5.0 suggests that PsbS adopts a more ordered structure at low pH, in line with the DOSY results, where fast proton-exchange effects were more prominent at pH 7.5 than at pH 5.0. Our observations match with the conclusion of Fan *et al*., that PsbS dimers form a more loose structure at neutral pH^18^. The supposed monomer spectrum at low pH (Fig. 8c, green) has several shifted peaks and lacks the Arg signal. *Physcomitrella patens* PsbS contains three Arg residues, of which one is involved in an inter-monomer hydrogen bond, stabilizing the PsbS dimer^18^. The disappearance of the Arg signal could correlate with breaking of the inter-dimer hydrogen bond, and the shifted peaks could be from sites at the monomer interfaces.

In conclusion, we observe that refolded *Physcomitrella patens* PsbS stabilizes as dimers over time in detergent solutions that are carefully kept above the critical micelle concentration (CMC), indicating that strong interactions between the PsbS monomers occur. The crystallographic structure of native PsbS shows a chlorophyll bound at the dimer interface, which may stabilize the dimers *in vivo* ^18^. The necessity of lipid or pigments for membrane protein oligomerization is a common motif and for instance, LHCII, which is homologue to PsbS, requires 1,2-dipalmitoyl-sn-glycero-3-phospho-rac-(1-glycerol) for its trimerization^25^. In our experiments however, PsbS dimers are observed in an *in vitro* assay, which excludes the necessity for native lipids or pigments to stabilize the inter-monomer interfaces. PsbS dimers are formed at neutral and low pH conditions, which is consistent with the observed low-pH crystal structure of a PsbS dimer. Lowering of the pH, corresponding with the active state of PsbS *in vivo*, however appears to destabilize the dimer states or their association into higher soluble aggregates, and shifts apparent monomer-dimer equilibria towards monomers. This finding not only explains the controversies about the oligomeric states of PsbS at low pH reported in different studies^13,17,18^, but also suggests a molecular-response model where the pH environment may control active and inactive populations of PsbS in a gradual manner. In membranes *in vivo*, other parameters like zeaxanthin could further control monomer-dimer or higher-state equilibria and shift those towards the active form. Our combined results show that upon lowering the pH, local changes occur in PsbS conformational structure and dynamics that are not accompanied by large changes in the secondary structure. PsbS adopts a more loose structure at neutral pH than at low pH while at the same time its dimeric form is more stabilized. PsbS proteins in detergent solutions adapt slightly different conformational structures depending on the micellar microenvironments. *In vivo*, this plasticity might be used by PsbS to associate with different interacting partners.

### Conclusion and outlook

We successfully developed a protocol for large-scale overproduction, refolding and dimerization of PsbS, opening routes for structural characterization of this important light-stress sensor protein. Solution NMR spectroscopy forms an elegant method to simultaneously analyze protein PsbS conformational structures and oligomerization states. Due to the large protein-micelle sizes, a thorough structural analysis will require solid-state NMR approaches that have the advantage that there is no size limit. The conformational structures of PsbS in detergent micelles are influenced by the choice of detergent, underlining the importance of the protein microenvironment. Our results reveal that PsbS has intrinsic properties to form dimers both at low and neutral pH, but does undergo pH-dependent conformational changes that destabilize the dimer states and shift monomer-dimer equilibria. In membranes *in vivo*, additional components may further control the equilibria to create a fine-tuned photoprotective response mechanism for PsbS activation upon thylakoid-lumen acidification at excess light conditions.

## Methods

### Cloning and overexpression of recombinant PsbS from *Physcomitrella patens*

To optimize recombinant *Physcomitrella patens* PsbS protein production the gene was inserted in pExp5-NT/TOPO^®^ (Invitrogen). Using the Gibson assembly technique, a PCR fragment containing the coding region of *Physcomitrella patens* PsbS from pETite-PsbS was inserted in frame with a N-terminal His-tag into pExp5-NT/TOPO^®^.

Overexpression of PsbS was carried out by inducing *E. coli* strain BL21(DE3) pLysS grown on Luria Bertani media containing 75 µg/mL ampicillin and 25 µg/mL chloramphenicol by addition of 0.05 mM of isopropyl-ß,D-thiogalactopyranoside (ITPG) at cell density of 0.3-0.4. After 12-14 hours at 18 °C, the culture was harvested. For NMR HSQC experiments, bacteria were grown in M9 minimal media along with ^15^NH_4_Cl. The cell pellet was stored at −80 °C until further use.

### Protein purification using a urea-wash protocol or nickel-affinity column

Purification of inclusion bodies was carried out using detergent buffer and Triton buffer as described in ref.^26^. Several washes of Triton buffer resulted in a white pellet, which was abundant in unfolded PsbS. Two different methods were compared for purification of PsbS from the pellet: nickel affinity column and urea wash. The latter method used a novel protocol that we designed to optimize the yield of purified PsbS. For His-Tag purification, a nickel-affinity column protocol was followed (Qiagen method to isolate denatured protein). At the end of this step, purified unfolded PsbS was obtained. The new protocol used UP buffer (8 M urea in 100 mM sodium phosphate, 100 mM Tris pH 8.0) to prevent the loss of protein during nickel affinity column purification. The inclusion body pellet was dissolved in urea buffer and incubated at 25 °C, 900 rpm shaking for 30 minutes. The sample was centrifuged at 20,000xg at room temperature for 10 minutes. This step produced a pellet containing PsbS and a soluble fraction containing impurities. The urea wash and centrifugation step were repeated twice. Washing was done with UP buffer and 0.05% LDS which further removed impurities. Next, the remaining PsbS pellet was dissolved in urea buffer by adding 0.5% of lithium dodecyl sulfate (LDS) which helped dissolve PsbS. Immediate buffer exchange (HiTrap Desalting, GE Healthcare) to 50 mM Hepes, 0.1% LDS, pH 5.0 was performed to remove the high concentration of urea and to adjust the pH. The recombinant PsbS in 50 mM Hepes, 0.1% LDS, pH 5.0 was stored at 4 °C until further use.

### Refolding of recombinant PsbS

Refolding of the LDS-solubilized PsbS was performed adapting the protocol from^19^, using various detergents purchased from Anatrace for refolding: n-Octyl-β-D-Glucopyranoside (OG), n-Dodecyl-β-D-Maltoside (DDM), Brij-78, n-Dodecylphosphocholine (FC-12), 1,2-Dihexanoyl-sn-Glycero-3-phospho choline (DHPC), n-Nonyl- β-D-Glucopyranoside (NG). The refolding buffer contained 0.1 M Hepes (adjusted to pH 5 or pH 7.5 with NaOH), 4% (wt/vol) LDS, and 25% (wt/vol) sucrose. Unfolded protein (~1 mg/mL) was mixed with an equal volume of refolding buffer and heated to 100 °C for 1 min. The desired detergent was added to the mixture and 200 mM KCl was used to precipitate LDS. Critical micelle concentration (*N*_*det,CMC*_), aggregation number (*N*_*det,Agg*_)^24^ of each detergent type and volume of sample was taken into account while calculating the percentage of detergent to add (Eq. 1).

1 $${N}_{det,tot}={N}_{det,CMC}+{N}_{det,Agg}[PsbS]$$

Finally, buffer exchange to 100 mM sodium phosphate for pH 7.5 or to 100 mM sodium acetate for pH 5.0 was carried out (HiTrap Desalting, GE Healthcare), to remove the excess sucrose. For NMR experiments, samples were concentrated using Amicon Ultra-4 centrifugal filters containing 10 kDa filter membranes.

### Gel electrophoresis and Western blotting

SDS-page gel electrophoresis analysis (12.5% running gel, 4% stacking gel stained with Coomassie brilliant blue R-250 Bio-Rad) was carried out for checking the yield of PsbS at every step of overexpression, purification and refolding. 2.5 µL of Precision Plus Protein™ Dual Color Standard from sigma was used in every SDS-page gel. As a control to check the presence of PsbS, western blotting was carried out using Anti-PsbS *Arabidopsis thaliana* (Agrisera antibodies AS09533) following Bio-Rad protocol. A Western Blot, confirming the presence of PsbS, is shown in Supplementary Fig. S7.

### Circular dichroism spectroscopy

The secondary folds of the refolded PsbS were analyzed by UV CD spectroscopy (Jasco J-815) using step size of 1 sec/nm, bandwidth of 1 nm and a scanning speed 100 nm/min. CD spectra were analyzed using BeStSel (Beta Structure Selection^27^) for estimating the content of α-helical, beta sheet and coil or other conformations for each CD spectrum.

### Dynamic Light Scattering

DLS was carried out at room temperature using a Malvern Zetasizer Nano S90 with VWR cuvettes PMMA semi micro.

### Size exclusion chromatography

SEC was carried out on Superdex 200 5/150 GL analytical column (GE Healthcare). The column was equilibrated with 100 mM sodium phosphate with 0.05% FC12 or 1% OG for pH 7.5 and with 100 mM sodium acetate with 0.05% FC12 or 1% OG for pH 5.0. All samples were filtered using 0.22 µm filters before loading on the column. The detection wavelength was set at 260 nm. The sizes of the eluted fractions were estimated with the use of molecular weight markers as shown in the Supplementary Materials section.

### NMR spectroscopy

Spectra were recorded on a Bruker Avance III 600-MHz instrument equipped with a cryoprobe. A standard 1D sequence with ^15^N decoupling during acquisition was used to obtain 1D ^1^H NMR data. For the 2D experiments, standard HSQC was performed with 2D H-1/X correlation via double inept transfer with decoupling during acquisition. The pulse program used for obtaining 1D ^1^H DOSY spectra was stebpgp1s19, which contains a 2D sequence for diffusion measurements using stimulated echo using bipolar-gradient based diffusion-ordered spectroscopy. For all the experiments, water signal was suppressed by using a 3-9-19 pulse sequence with gradients. The diffusion time interval (d20 or Δ) was set to 300 ms while the gradient pulse lengths (p30 or δ) was set to 2 ms for all the measurements. The pulse gradient field strength was incremented linearly from 2% to 95% in 16 steps. The 90° pulse for proton was found to be 11.8 μs at −7.00 dB and 64 scans were used with a relaxation delay of 8 s. The DOSY intensity curves (*I)* plotted versus the gradient strength (*g*) were fitted with a double-exponential fit according to Eq. (2) to obtain the translational diffusion constant *D*_*t*_ using an Igor-Pro user-defined fit function (Igor Pro version 6.01):

2 $$I({D}_{1,t},{D}_{2,t},g)={I}_{1}{e}^{-{D}_{1,t}^{2}{\gamma }^{2}{g}^{2}{\delta }^{2}[{\rm{\Delta }}-\frac{1}{3}\delta ]}+{I}_{2}{e}^{-{D}_{2,t}^{2}{\gamma }^{2}{g}^{2}{\delta }^{2}[{\rm{\Delta }}-\frac{1}{3}\delta ]}$$

The resulting fits yielded two diffusion constants *D*_1_ and *D*_2_ in the order of ~10^−9^ m^2^/s and ~10^−11^ m^2^/s, respectively. *D*_1_ was ascribed to the effects of chemical exchange, while *D*_2_ was taken as the translational diffusion constant *D*_*t*_. Particle hydrodynamic sizes were calculated from *D*_*t*_ using a viscosity constant *η* = 0.88 c_p_, which value was also used for calculation of the DLS-determined hydrodynamic sizes.

## Electronic supplementary material

Supplementary Information
