# Supplementary material for: Large-scale in vitro production, refolding and dimerization of PsbS in different microenvironments
Source: Sci Rep. 2017 Nov 9;7:15200. doi: 10.1038/s41598-017-15068-3 (PMC5680255; doi:10.1038/s41598-017-15068-3)
Supplement: Supplementary file 1 — Supplementary Information [file 41598_2017_15068_MOESM1_ESM.pdf]

Supplementary Information

Large-scale *in-vitro* production, refolding and dimerization of PsbS in different microenvironments

Maithili Krishnan[1], Geri Moolenaar[1], Karthick Sai Sankar Gupta[1], Nora Goosen[1], and Anjali Pandit[1]*

[1]Leiden University, Leiden Institute of Chemistry, Gorlaeus Laboratories, Einsteinweg 55
2333 CC Leiden, The Netherlands
*corresponding author : a.pandit@lic.leidenuniv.nl

Figure S1: Inclusion body purification from 200 mL cell pellet.

(+) IPTG sample shows the overexpression of PsbS from 200 mL culture. Several washes of inclusion body purification were carried out (supernatant S1 to pellet P4). Bovine serum albumin protein (1.5 µg) is shown for comparison of yield. In the final step, pellet P4 contains PsbS of ~19mg, which was used for the next step, urea wash purification.

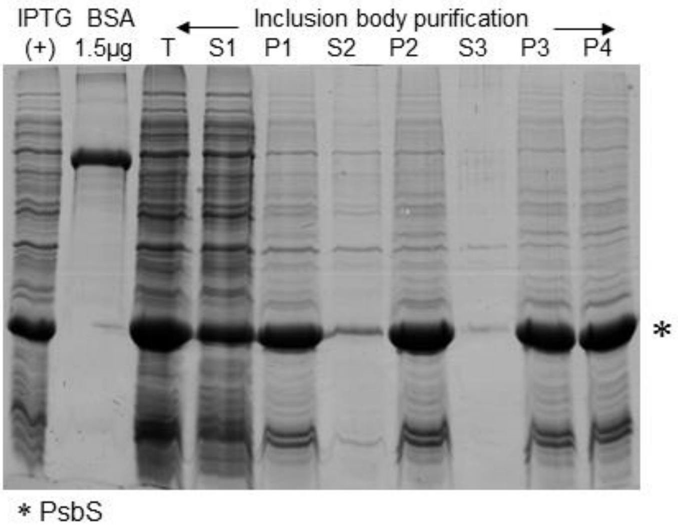

Figure S2 Purification of Lhcb1 (*Arabidopsis thaliana*) from inclusion body pellet using the urea wash protocol.

Lane 1: Lhcb1 from the inclusion bodies pellet was dissolved in buffer containing 8M urea. Several washes of urea buffer were carried out (Lane 2,3,4). Lane 5 is washing step of pellet with 8M urea buffer with 0.05% Lithium dodecyl sulfate (LDS). The last wash step was carried out using urea buffer with 0.5% of LDS to dissolve all the PsbS from inclusion bodies (Lane 6). Lane 7 contains 3µg of bovine serum albumin for yield comparison.

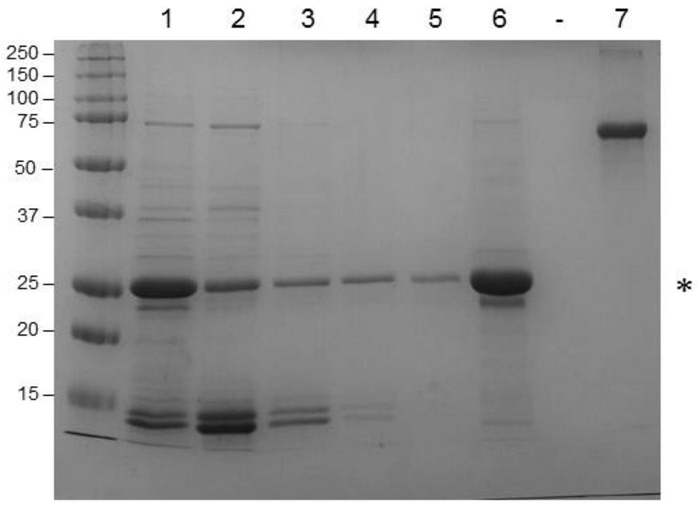

* Lhcb1

Figure S3 Homology model of dimeric PsbS from *Physcomitrella patens*

A homology model of *Physcomitrella patens* PsbS constructed by SWISS-MODEL based on the crystal structure of PsbS from *Spinacia oleracea* (PDB-ID 4RI2). The homology structure includes the stromal loops that are not resolved in the crystal structure and was energy-minimized using Chimera software[1]. (a) front view of *Physcomitrella patens* PsbS (b) side view of *Physcomitrella patens* PsbS.

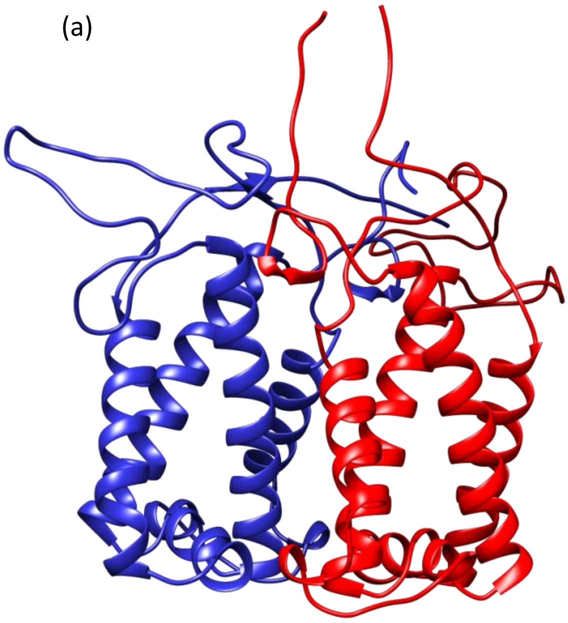

Figure S4: Boiled (B) and unboiled (UnB) samples of PsbS in FC 12.

Using the standard (sodium dodecyl sulfate) SDS-page gel protocol including sample boiling, both monomer and dimer bands of PsbS are observed in a 90-days old sample. If the boiling step before loading is omitted, only dimer bands are observed.

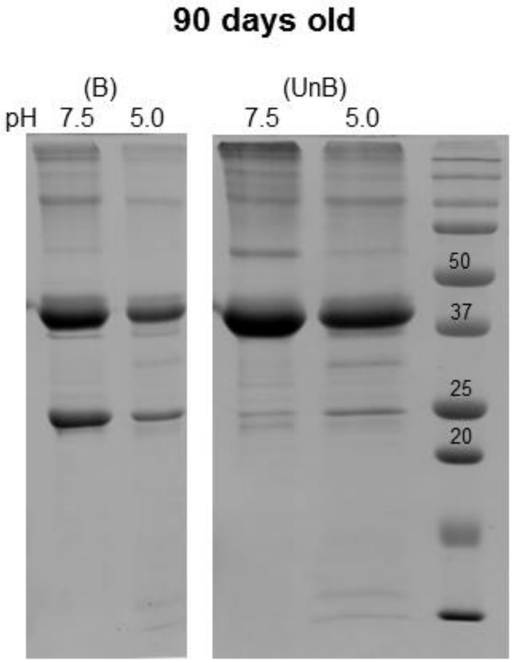

Figure S5: Comparison of size exclusion chromatograms of PsbS in *n*-Octyl-*β*-D-Glucopyranoside (OG) at pH 7.5 with the detection wavelength set at 260 nm (solid), and at 214 nm (dash) detection.

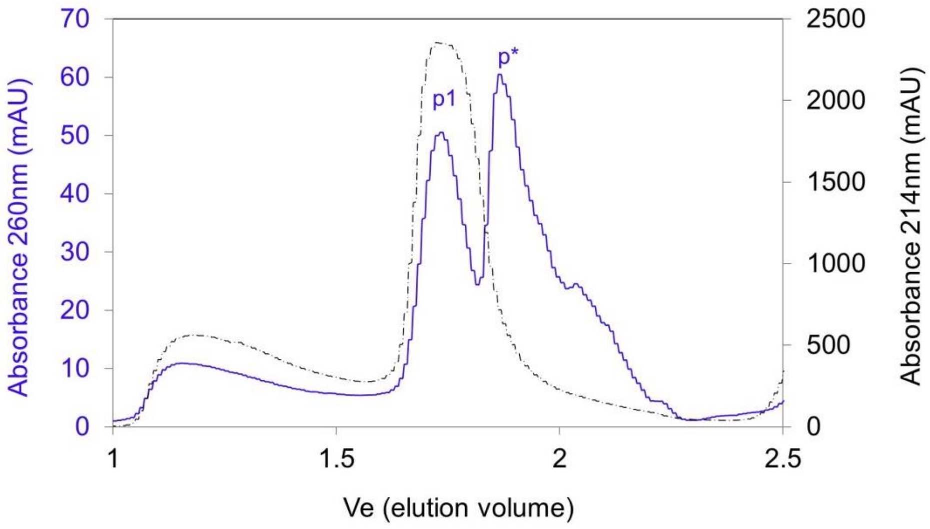

Table S1: Molecular weight estimates for SEC eluted fractions of PsbS in *n*-Dodecyl phosphocholine (FC-12)

Sample Description	*Ve*	*MW (kDa)*	*Exp*	*Y*
*PsbS pH 7.5 peak **	*1.92*	***50***	*0.00135*	*50.6531*
PsbS pH 7.5 peak 1	*1.75*	***90***	*0.00243*	*90.9036*
FC-12 detergent	*1.98*	***41***	*0.001101*	*41.2066*
*PsbS pH 5.0 peak **	*1.95*	***45***	*0.00122*	*45.6864*
PsbS pH 5.0 peak 1	*1.65*	***128***	*0.003427*	*128.226*

Table S2 Molecular weight estimations for SEC eluted fractions of PsbS in OG

Sample description	*Ve*	*MW (kDa)*	*Exp*	*Y*
*PsbS pH 7.5 peak **	*1.87*	***60***	*0.00186*	*60.6579*
PsbS pH 7.5 peak 1	*1.72*	***100***	*0.003081*	*100.439*
OG detergent	*1.92*	***51***	*0.001573*	*51.2722*
*PsbS pH 5.0 peak **	*1.89*	***56***	*0.00174*	*56.7134*
PsbS pH 5.0 peak 1	*1.78*	***82***	*0.002518*	*82.0911*

Figure S6 Calibration curve in FC12 (a) and in OG (b).

Markers 1, 2, 3 and 4 correspond to α-amylase (200KDa), alcohol dehydrogenase (150KDa), bovine serum albumin (66KDa) and carbonic anhydrase (29KDa) (green diamond).

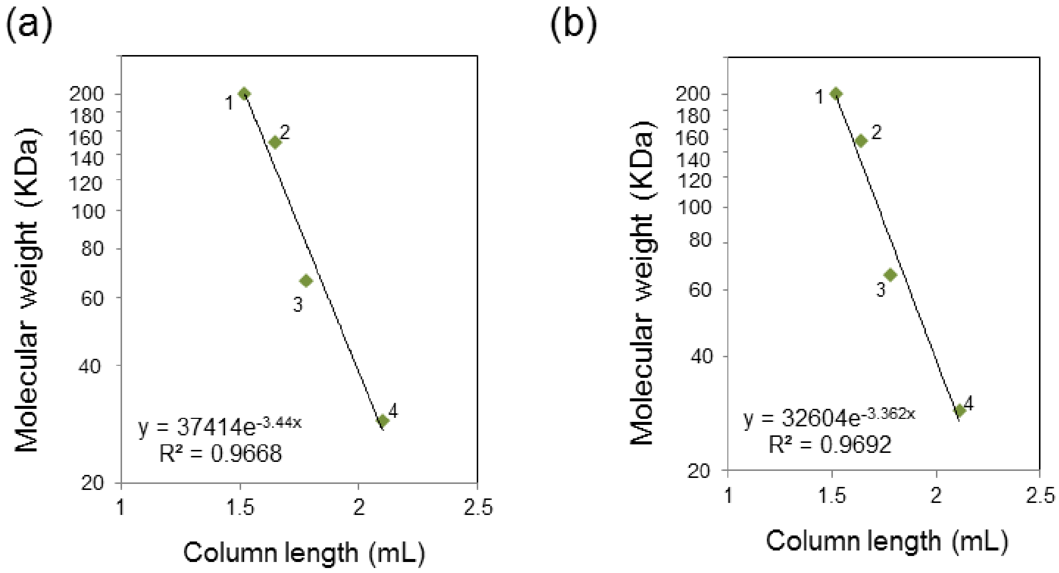

Fig S7: Western blot of refolded PsbS using Anti-PsbS (Agrisera antibodies AS09533)

(a) SDS-page gel containing PsbS refolded in FC-12.
(b) Refolded PsbS was run on western blot using Anti-PsbS antibodies to detect the presence of PsbS protein. The presence of dimer, monomer and higher aggregates are observed.

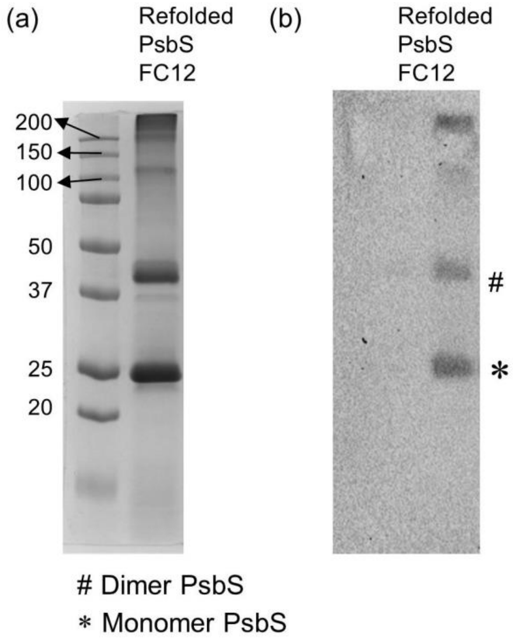

Fig S8: Original SDS-page gel figure graphs that were presented in edited form (cutting two lanes) in Figure 4 in the main text.

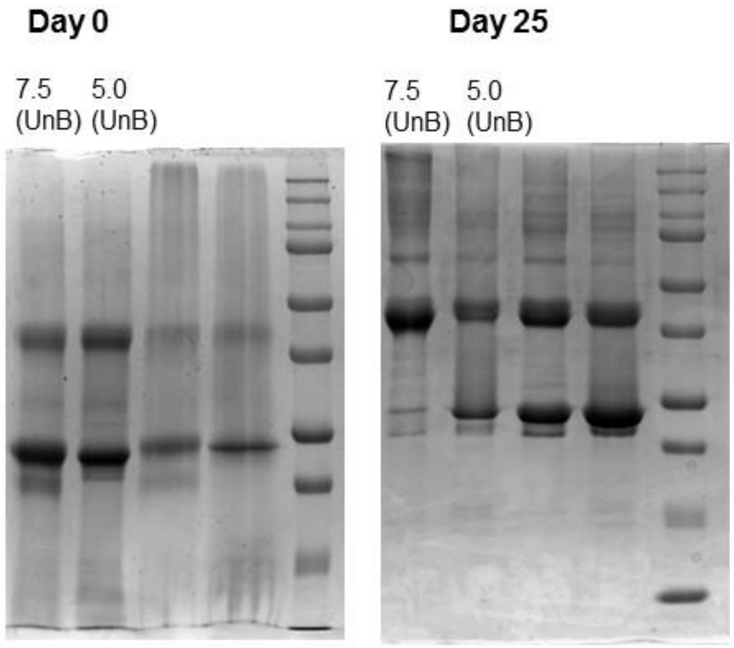

References

1. Pettersen, E. F. *et al.* UCSF Chimera — A Visualization System for Exploratory Research and Analysis. (2004). doi:10.1002/jcc.20084
